# Effect of acupuncture combined with antidepressants on post-stroke depression: A network meta-analysis of nine acupuncture therapy

**DOI:** 10.3389/fneur.2023.979643

**Published:** 2023-03-23

**Authors:** Chun Li, Shasha Chen, Shuang Liu, Yang Mu, Mouxiao Su

**Affiliations:** ^1^Department of Rehabilitation Medicine, Mianyang Central Hospital, School of Medicine, University of Electronic Science and Technology of China, Mianyang, Sichuan, China; ^2^Department of Rehabilitation Medicine, Chongqing University FuLing Hospital, Chongqing, China

**Keywords:** acupuncture, antidepressants, post-stroke depression, network meta-analysis, evidence

## Abstract

**Background:**

Post-stroke depression (PSD) is a common psychiatric complication of mental disorders after stroke. Acupuncture for PSD is effective and has few adverse effects. As a classical complementary and alternative therapy, acupuncture is often used in combination with antidepressants for PSD. However, there is a wide variety of acupuncture therapies, and the efficacy of different acupuncture varies. In this study, a network meta-analysis (NMA) was used to assess the clinical efficacy of different acupuncture combined with antidepressants for the treatment of PSD.

**Methods:**

A comprehensive search of PubMed, The Cochrane Library, EMbase, Web of Science, CNKI, CBM, VIP, and Wan-Fang databases for published randomized controlled trials of acupuncture combined with antidepressants for the treatment of PSD was conducted. The time frame for the literature search was from the date of database creation to April 30, 2022. The Cochrane risk of bias tool for randomized trials (RoB 2.0) was used to evaluate the bias risk of the included studies. Data analysis was performed by STATA 14.0 software.

**Results:**

A total of 38 literatures with 2,898 patients involving nine acupuncture therapies were included. NMA results were as follows: moxibustion plus antidepressants had the best efficacy in terms of improving total effective rate. Conventional acupuncture plus antidepressants was the most effective in improving HAMD scores. In terms of improving SDS scores, acupressure plus antidepressants was the most effective. In terms of improving NIHSS scores, moxibustion plus antidepressants showed the best results.

**Conclusion:**

A comparison of the efficacy indicators of the nine different acupuncture therapies combined showed that moxibustion plus antidepressants, conventional acupuncture plus antidepressants and acupressure plus antidepressants were superior in the treatment of PSD. Based on the shortcomings of the existing studies, this conclusion needs to be validated by additional high-quality randomized controlled trials.

## 1. Introduction

Post-stroke depression (PSD) is a psychological disorder characterized by persistent depression, decreased interest and behavioral withdrawal after stroke (1, 2). The main manifestations are psychiatric symptoms, such as insomnia, depression and low self-esteem, and even suicidal tendencies, which can lead to cognitive impairment (3, 4). Nosologically, PSD is a special type of depression that lacks clear concept and diagnostic criteria at present. In clinical practice, PSD is mainly diagnosed based on the history of stroke, manifestations of depression, and auxiliary assessment of relevant scales. The International Classification of Diseases, 10th Edition (ICD-10) classifies PSD as an organic mood disorder. The American Diagnostic and Statistical Manual of Mental Disorders, 5th Edition (DSM-V) classifies PSD as a depressive disorder due to other physical disorders. The 3rd edition of Chinese Criteria for Classification and Diagnosis of Mental Disorders (CCMD-3) classifies PSD as a mental disorder caused by cerebrovascular diseases. Related studies have shown that the prevalence of PSD is as high as 30–35% (5). Within 2 months of a stroke, 36% of patients present clinical manifestations of depression, involving 14% who are diagnosed with major depression (6). The incidence of PSD increases gradually with the prolongation of the course of disease. The high prevalence of PSD not only increases the mental suffering of patients and reduces their quality of life, but also brings a heavy burden to families and society (7). Clinical symptoms of definitely diagnosed cases of PSD can be relieved to yield the clinical cure. The disability rate and suicide rate can be significantly minimized, and the quality of life and social function are remarkably improved, thus preventing the relapse. Therefore, it is important to reduce PSD in stroke survivors.

At present, the main drugs for clinical treatment of PSD are monoamine oxidase inhibitors, tricyclic antidepressants, selective 5-hydroxytryptamine reuptake inhibitors and new antidepressants developed in recent years (8–10). The use of antidepressants may be appropriate for both major and minor depression, although the optimal use of which antidepressants has not been recommended by international guidelines (11, 12). Recent studies have shown that escitalopram is associated with a faster relief from depression, and Mirtazapine may be the best option (13). Although antidepressants are the main clinical treatment for PSD, there are still many adverse effects, drug interactions, and drug resistance in patients taking medication for a long time (14, 15). Acupuncture, as a characteristic therapy of Chinese medicine, has achieved positive results in the treatment of PSD with remarkable efficacy and low adverse effects (16).

Acupuncture is often used in combination with antidepressants for PSD. Studies have confirmed that acupuncture combined with antidepressants is superior to antidepressants alone (17–21). Huang’s study showed that electroacupuncture combined with antidepressants was satisfactorily effective in treating PSD, relieving the patients’ depressive symptoms and improving their sleep quality (22). Liu’s study showed that moxibustion combined with antidepressants for PSD was effective in relieving patients’ depressive symptoms and promoting neurological recovery (23). Zhou et al. showed that acupuncture combined with antidepressants improved the depressive status of PSD patients and showed varying degrees of improvement in motor function, activities of daily living, and the degree of neurological deficits in PSD patients (24). A relevant meta-analysis also showed that acupuncture combined with antidepressants is more effective in treating PSD than the monotherapy of antidepressants (25, 26). In terms of the treatment time of acupuncture and moxibustion, Since PSD is a chronic disease, most studies recommended that the treatment course of PSD by acupuncture should be longer than 6 weeks, and the curative effect is positively correlated with the treatment course. Controversially, short-term benefits of less than 6 weeks have also been reported. However, there is a wide variety of acupuncture therapies, including conventional acupuncture, moxibustion, electroacupuncture, warm acupuncture, auricular acupuncture, fire acupuncture, etc. A direct comparison of the efficacy of different acupuncture therapies combined with antidepressants is lacking. Therefore, in the real world, the choice of which acupuncture therapy should be combined with antidepressants remains controversial. So far, direct comparative studies analyzing different therapies are limited. Network meta-analysis (NMA) ranks the effectiveness of different interventions by combining direct and indirect evidence and further synthesizing the results of direct and indirect comparisons, which is important for clinicians to make the best treatment choices (27). Therefore, this study used the NMA method to compare the efficacy of different acupuncture combined with antidepressants for the treatment of PSD, and to provide a basis for choosing the best combination protocol for the clinical treatment of PSD.

## 2. Methods

NMA was performed in accordance with the Preferred Reporting Items for Systematic Reviews and Meta-Analyses (PRISMA) guidelines (28).

### 2.1. Inclusion criteria

#### 2.1.1. Type of clinical research

A randomized controlled trial (RCTs) of acupuncture combined with antidepressants versus antidepressants for PSD will be included. The publish language is limited to English and Chinese.

#### 2.1.2. Participants

Included patients were diagnosed with PSD and were not limited in gender, race, comorbidity, or duration of disease.

#### 2.1.3. Interventions and comparisons

The control group used antidepressants alone. The experimental group used acupuncture combined with antidepressants, and acupuncture-related therapies included conventional acupuncture, moxibustion, electroacupuncture, warm acupuncture, ear acupuncture, fire acupuncture, etc.

#### 2.1.4. Outcomes

The primary outcome indicators were total effective rate and Hamilton Depression Scale (HAMD) score. If the HAMD score decreased by more than 75% compared with that before treatment, it showed that it was a cure; if the HAMD score decreased by 50–75% compared with that before treatment, it showed an obvious effect; if the HAMD score decreased by 25–49% compared with that before treatment, it showed an effective result; if the HAMD score decreased by less than 25% compared with that before treatment, it showed that it was ineffective. Total effective rate = cure rate + obvious effective rate + effective rate (29).

Secondary outcome indicators were National Institutes of Health Stroke Scale (NIHSS) and Depression Self-Rating Scale (SDS) scores. The included literature had clear and relevant evaluation criteria.

### 2.2. Exclusion criteria

(1) Duplicate published literature.
(2) No clear diagnostic criteria and no efficacy criteria.
(3) Literature with incorrect data or inaccessible data.

### 2.3. Search strategy

A comprehensive search of PubMed, The Cochrane Library, EMbase, Web of Science, CNKI, CBM, VIP, and Wan-Fang databases for published randomized controlled trials of acupuncture combined with antidepressants for the treatment of PSD was conducted. The time frame for the literature search was from the date of each database creation to April 30, 2022. MeSH terms were paired with free terms, using the appropriate Boolean logical operator connections for the search, and appropriate adjustments were made according to the different databases to include all retrieved literature. For Pubmed, for example, the specific search strategy is described in Supplementary Table S1.

### 2.4. Data extraction

The literature was screened, extracted and cross-checked by 2 investigators independently. In case of disagreement, the decision was made by the corresponding author. For literature screening, the title of the text was read first, and after excluding apparently irrelevant literature, the abstract and full text were further read to determine inclusion. Enter the data needed by this research by excel. Data was extracted from: (i) basic information of the included studies, including study title, authors, year and month of publication, sample size, and diagnostic criteria; (ii) interventions, including types and duration of antidepressants and acupuncture; (iii) characteristics of study subjects; (iv) evaluation entries and results of risk of bias evaluation; and (v) outcome indicators, including total effective rate, HAMD score, NIHSS score, and SDS score.

### 2.5. Quality assessment

Risk of bias evaluation of the included RCTs by 2 evaluators according to the Cochrane risk of bias tool for randomized trials (RoB 2.0) (30). Items evaluated included randomization process, deviations from the intended interventions, bias for missing outcome data, bias for outcome measures, and bias for selective reporting of outcomes. All entries were rated as “low risk,” “high risk,” or “some concerns.” Trials were rated as “low risk” overall only if all items were assessed as “low risk.” As long as there was a high risk of bias in one of all items, the trials had a high risk of bias. In addition to the above two cases, the risk of trial bias was uncertain.

### 2.6. Data analysis

Dichotomous variables were expressed using relative risk ratio (RR) and its 95% confidence interval (95% CI). Continuous variables were expressed using standardized mean difference (SMD) and its 95% CI. The *χ*^2^-test evaluated the heterogeneity between the results of the included studies, and *I*^2^ quantified the heterogeneity. When *p* ≥ 0.1 and *I*^2^ < 50%, homogeneity was indicated and a fixed-effects model was selected for analysis; conversely, a random-effects model was used. Subgroup analysis according to treatment duration (>6 weeks an*d* ≤ 6 weeks). Sensitivity analysis to test the stability of meta-analysis results. If no less than 10 papers were included, small sample effects or publication bias were detected by comparison-adjusted funnel plots. Since this study is an indirect comparison of acupuncture combined with antidepressants versus antidepressants for PSD, no consistency test is required. Statistical analysis was performed using Stata 14.0 software and its network, mvmeta package, etc., to draw a net relationship diagram, funnel diagram, etc. for comparison between interventions. The effects of the interventions were ranked by surface under the cumulative ranking curve (SUCRA); larger SUCRA values were ranked higher, indicating that the intervention was more likely to be the most successful one.

## 3. Results

### 3.1. Literature screening result

**Figure 1:**
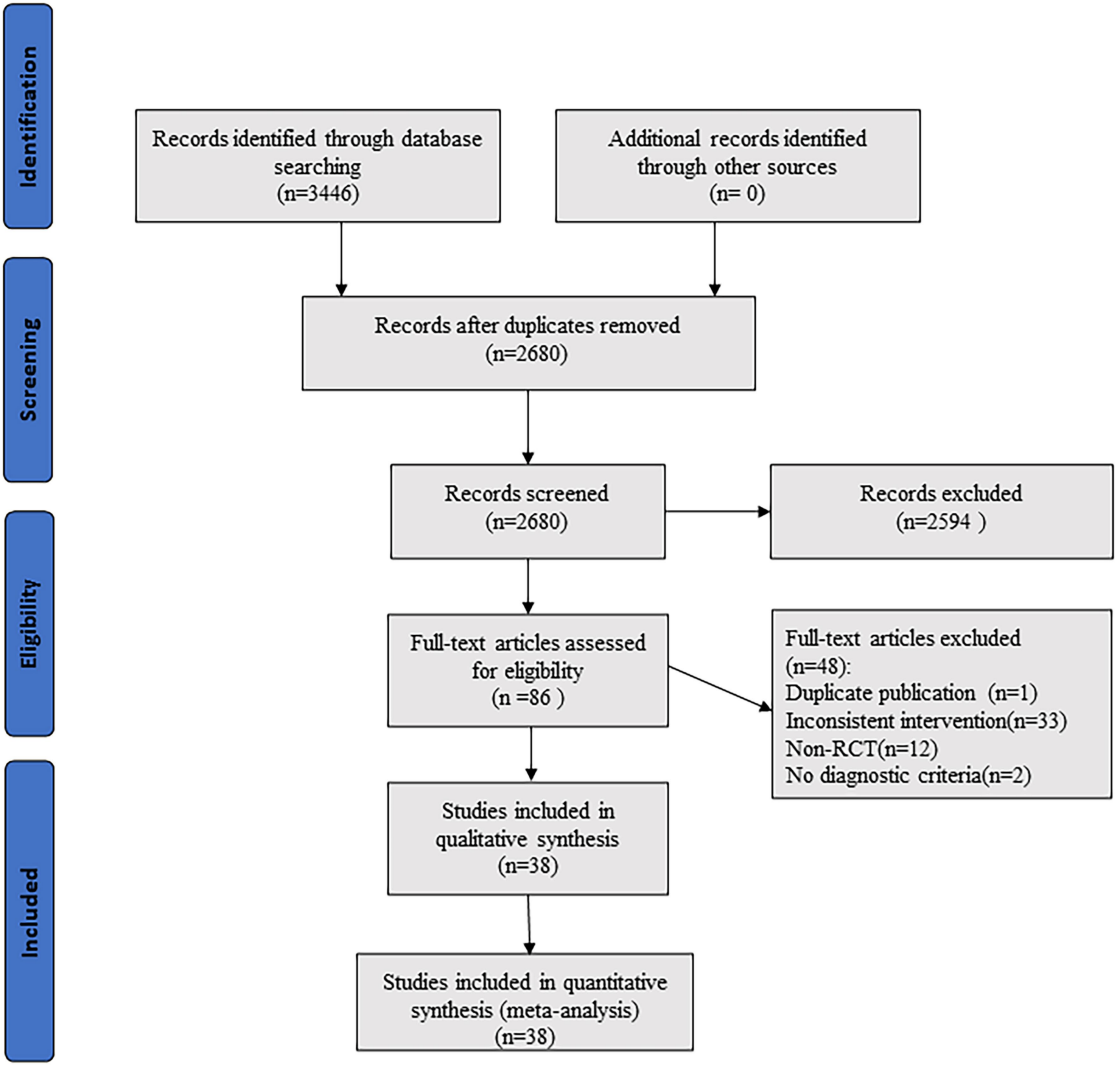
Literature screening process.

After initial review of 3,446 relevant literature, 38 literatures with 2,898 patients were finally included after stratum-by-stratum screening. The literature screening process and results are shown in Figure 1.

### 3.2. Characteristics of included literature

A total of 2,898 patients with 38 literatures (17–24, 31–60) involving nine acupuncture treatments were included in this study. The acupuncture therapies included: wrist-ankle acupuncture (an acupuncture therapy in which subcutaneous acupuncture is taken from the corresponding points of the wrist and ankle to treat diseases), moxibustion (a therapeutic method for preventing and curing diseases by stimulating acupoints or specific parts of human body by stimulating the activities of meridian qi to adjust the physiological and biochemical functions of human body disorders and thus achieving the purpose of preventing and curing diseases by burning moxa sticks or moxa columns made of moxa leaves), electroacupuncture (a therapeutic method in which a needle is inserted into a certain part of the human body and then an electric current is applied to the needle), conventional acupuncture (using filiform needles as needling tools and using different manipulations to stimulate meridians and acupoints of human body, it is a method to dredge meridians, harmonize qi and blood, adjust viscera functions and treat diseases), auricular electroacupuncture (a treatment method combining traditional auricular acupuncture with pulse current stimulation), auricular pressure (according to the principle of “ear acupuncture,” small plant seeds are applied to stimulate acupoints and reaction points on auricle to prevent and cure diseases), acupressure (pressing specific acupoints of human body stimulates the qi of meridians to achieve the purpose of dredging meridians, regulating human function, eliminating pathogenic factors and strengthening body resistance), abdominal acupuncture (a brand-new acupuncture therapy for treating systemic diseases by acupuncture at specific acupoints in abdomen to adjust qi activity, yin and yang and realize the dynamic balance of yin and yang in human body) and eye acupuncture (acupuncture treatment of systemic diseases by acupuncture at acupoints around the eyeball and the edge of the orbit). There are seven diagnostic criteria for PSD. Antidepressants included fluoxetine, duloxetine, flupentixol and melitracen tablets, citalopram, paroxetine, sertraline, St. John’s wort extract, venlafaxine and mirtazapine. The course of treatment is 2–13 weeks. Basic characteristics of the included literature are shown in Table 1.

**Table 1:** Characteristics of included studies.

Included studies	Diagnostic criteria	Sample		Age		Interventions		Treatment course (week)	Outcome indicators
		T	C	T	C	Intervention of treatment	Intervention of control		
You Yanli 2020	①	66	33	30 ~ 75	30 ~ 75	WAA + Fluoxetine	Fluoxetine	4	HAMD, SDS
Zhang Ting 2016	②	29	27	64.51 ± 9.12	63.74 ± 8.79	Moxibustion + Fluoxetine	Fluoxetine	8	Total effective rate
Liu Songlin 2019	③	35	35	58.38 ± 10.65	59.82 ± 10.41	Moxibustion + Duloxetine	Duloxetine	4	HAMD, NIHSS
Huang Shile 2014	②	30	30	62.77 ± 9.32	62.10 ± 8.11	Electroacupuncture + Fluoxetine	Fluoxetine	6	HAMD,Total effective rate
Liu Yuhong 2016	②	43	43	57 ~ 74	57 ~ 74	Electroacupuncture + Flupentixol and Melitracen Tablets	Flupentixol and Melitracen Tablets	4	HAMD, NIHSS
Guo Aisong 2011	②	32	31	40 ~ 65	40 ~ 65	Electroacupuncture + Fluoxetine	Fluoxetine	6	HAMD
Sun Baomin 2010	⑦	36	37	72.2 ± 11.2	71.9 ± 10.8	Electroacupuncture + Citalopram	Citalopram	4	HAMD
Peng Huiyuan 2009	②	30	30	50 ~ 80	50 ~ 80	Electroacupuncture + Fluoxetine	Fluoxetine	4	SDS
Li Li 2011	②	20	19	65 ± 13	63 ± 12	Electroacupuncture + Paroxetine	Paroxetine	4	HAMD
Zhang Rui 2017	②	35	35	35 ~ 75	35 ~ 75	Electroacupuncture + Paroxetine	Paroxetine	4	HAMD, NIHSS, Total effective rate
Jiao Haifeng 2012	②	17	16	43 ~ 78	45 ~ 76	Electroacupuncture + Paroxetine	Paroxetine	4	HAMD, Total effective rate
Wang Zhonghua 2008	②	40	40	56.7 ± 9.5	57.2 ± 9.1	Electroacupuncture + Fluoxetine	Fluoxetine	4	HAMD, Total effective rate
Tong Xin 2012	④	30	30	58.5 ± 8.4	58.5 ± 8.4	Conventional acupuncture + Fluoxetine	Fluoxetine	8	HAMD, Total effective rate
JiaoYonggang 2018	⑦	49	48	60.8 ± 7.2	61.3 ± 7.2	Auricular Electroacupuncture + Fluoxetine	Fluoxetine	6	HAMD, Total effective rate
Shao Hongwei 2020	⑦	40	40	54.36 ± 6.31	54.64 ± 8.21	Auricular Electroacupuncture + Fluoxetine	Fluoxetine	6	HAMD, Total effective rate
Duan Jun 2022	⑦	30	30	57.26 ± 17.31	58.38 ± 18.45	Auricular pressure + Sertraline	Sertraline	2	HAMD, SDS, Total effective rate
Zhang Yongchao 2020	②	58	57	59.24 ± 7.24	58.92 ± 6.27	Acupressure + Flupentixol and Melitracen Tablets	Flupentixol and Melitracen Tablets	8	SDS
Bi Xueqi 2017	⑦	20	20	71.2 ± 1.3	72.3 ± 1.9	Abdominal Acupuncture + Fluoxetine	Fluoxetine	4	HAMD, NIHSS, Total effective rate
Zhang Ekeng 2016	⑦	28	28	56.64 ± 8.32	57.32 ± 8.06	Abdominal Acupuncture + Fluoxetine	Fluoxetine	4	HAMD, NIHSS, Total effective rate
Wang Jingxin 2019	②	38	38	64.45 ± 4.48	64.59 ± 6.60	Conventional acupuncture + Paroxetine	Paroxetine	8	HAMD, Total effective rate
Liu Tai 2016	②	48	47	64.60 ± 10.35	65.38 ± 9.72	Conventional acupuncture + Paroxetine	Paroxetine	4	HAMD, Total effective rate
Zhang Shengli 2016	⑤	30	30	57.69 ± 6.9	56.81 ± 7.36	Electroacupuncture + Citalopram	Citalopram	6	HAMD, Total effective rate
Xu Jinping 2008	⑥	42	38	50 ~ 78	52 ~ 80	Eye acupuncture + Fluoxetine	Fluoxetine	8	Total effective rate
Huang Chunyuan 2013	②	80	76	61.10 ± 10.12	55.72 ± 9.02	Eye acupuncture + St. John’s wort extract	St. John’s wort extract	8	HAMD, Total effective rate
Zhang Qian 2015	②	30	30	59.4 ± 8.2	61.7 ± 8.4	Conventional acupuncture + Fluoxetine	Fluoxetine	4	HAMD, NIHSS
Zhou Yafen 2014	②	75	72	64 ± 15	65 ± 11	Conventional acupuncture + Fluoxetine	Fluoxetine	8	HAMD, NIHSS
Duan Xiaojing 2012	②	30	30	58.80 ± 9.60	60.22 ± 8.12	Conventional acupuncture + Flupentixol and Melitracen Tablets	Flupentixol and Melitracen Tablets	8	HAMD, Total effective rate
Jiang Lan 2019	②	27	27	55.6 ± 4.3	56.8 ± 5.2	Conventional acupuncture + Flupentixol and Melitracen Tablets	Flupentixol and Melitracen Tablets	4	HAMD, Total effective rate
Xu Changmin 2019	②	50	50	65.5 ± 1.4	65.2 ± 1.3	Conventional acupuncture + Paroxetine	Paroxetine	4	HAMD, Total effective rate
Li Chuanyou 2021	⑦	45	45	51.6 ± 8.4	53.5 ± 9.4	Conventional acupuncture + Citalopram	Citalopram	8	HAMD
Jian Rui 2020	⑦	44	43	64.6 ± 5.1	63.8 ± 4.3	Conventional acupuncture + Sertraline	Sertraline	8	HAMD, NIHSS, Total effective rate
Zhang Xiaodong 2019	②	42	42	62.35 ± 6.62	62.58 ± 6.84	Conventional acupuncture + Paroxetine	Paroxetine	6	HAMD, Total effective rate
Yan Changchang 2018	⑦	50	50	62.1 ± 13.5	62.3 ± 13.6	Conventional acupuncture + Venlafaxine	Venlafaxine	4	HAMD, NIHSS, Total effective rate
Han Yuhui 2018	②	67	67	59.83 ± 5.46	60.31 ± 5.27	Conventional acupuncture + Mirtazapine	Mirtazapine	8	HAMD, NIHSS, Total effective rate
Qi Linjing 2019	②	30	30	61.47 ± 7.42	61.52 ± 7.36	Conventional acupuncture + Sertraline	Sertraline	13	HAMD, NIHSS, Total effective rate
Xiao Wei 2011	②	30	30	50.62 ± 7.84	51.45 ± 6.28	Conventional acupuncture + Fluoxetine	Fluoxetine	8	HAMD, Total effective rate
Liu Dan 2013	②	20	18	42 ~ 68	42 ~ 68	Conventional acupuncture + Fluoxetine	Fluoxetine	4	Total effective rate
Zhu Fengkui 2010	②	30	30	DU	DU	Conventional acupuncture + Flupentixol and Melitracen Tablets	Flupentixol and Melitracen Tablets	4	Total effective rate

①, Hamilton Depression Scale; ②, Classification and Diagnostic Criteria of Mental Disorders in China-Third-Edition; ③, Handbook of neuropsychological Assessment scales for Dementia and Cognitive Impairment; ④, Diagnostic and Statistical Manual of Mental Disorders-Fifth-Edition; ⑤, International Classification of diseases- Tenth Edition; ⑥, Classification and Diagnostic Criteria of Mental Disorders in China-Second-Edition; ⑦, Expert consensus; SDS, Self-rating depression scale; HISSH, National Institute of Health stroke scale; HAMD, Hamilton Depression Scale; C, Control group; T, Treatment group; WAA, Wrist-ankle acupuncture.

### 3.3. Results of the risk of bias

10.52% of studies had low risk of bias, 13.16% of studies had high risk of bias and the remaining 76.32% had unclear risk of bias (Supplementary Table S2). The main causes of bias are inadequate randomization process and allocation hiding and lack of accurate information about blindness.

### 3.4. Pairwise meta-analysis

#### 3.4.1. Total effective rate

The results of the meta-analysis showed that electroacupuncture plus antidepressants, abdominal acupuncture plus antidepressants, auricular electroacupuncture pl-us antidepressants, conventional acupuncture plus antidepressants and auricular pressure plus antidepressants were statistically significant differences in total effective rate compared with antidepressants (*p* < 0.05). The results of descriptive analysis showed that the total effective rate of moxibustion plus antidepressants was statistically significant compared to antidepressants (*p* < 0.05). While the total effective rate of auricular pressure plus antidepressants compared with antidepressants, the difference was not statistically significant (*p* > 0.05). The results of pairwise meta-analysis are shown in Table 2.

**Table 2:** Pairwise comparisons of efficacy in RCTs.

Treatment 1	Treatment 2	No. of comparisons	Heterogeneity test		Meta-analysis		
			*I* ^2^	*P*	RR (95%CI)	SMD (95% CI)	*P*
Total effective rate							
Electroacupuncture + antidepressants	Antidepressants	5	0%	0.918	**1.19 (1.07, 1.32)**	**–**	**0.002**
Abdominal acupuncture + antidepressants	Antidepressants	2	0%	0.707	**1.14 (1.01, 1.28)**	**–**	**0.031**
Auricular electroacupuncture + antidepressants	Antidepressants	2	0%	0.886	**1.21 (1.06, 1.39)**	**–**	**0.006**
Auricular pressure + antidepressants	Antidepressants	1	**–**	**–**	1.29 (0.00, 1.67)	**–**	0.061
Conventional acupuncture + antidepressants	Antidepressants	14	4.30%	0.403	**1.19 (1.12, 1.25)**	**–**	**<0.0001**
Auricular pressure + antidepressants	Antidepressants	2	0%	0.572	**1.15 (1.02, 1.28)**	**–**	**0.02**
Moxibustion + antidepressants	Antidepressants	1	**–**	**–**	**1.30 (1.03, 1.65)**	**–**	**0.026**
Hamilton Depression Scale score							
Wrist-ankle acupuncture + antidepressants	Antidepressants	1	**–**	**–**	**–**	**−1.20 (−1.65, −0.75)**	**<0.0001**
Electroacupuncture + antidepressants	Antidepressants	9	86.90%	<0.0001	–	**−0.85 (−1.40, −0.30)**	**0.002**
Abdominal acupuncture + antidepressants	Antidepressants	2	0%	0.993	–	**−1.50 (−1.96, −1.05)**	**<0.0001**
Auricular electroacupuncture + antidepressants	Antidepressants	2	95.20%	<0.0001	–	−0.73 (−2.16, 0.70)	0.318
Auricular pressure + antidepressants	Antidepressants	1	–	–	–	**−0.56 (−1.08, −0.04)**	**0.033**
Conventional acupuncture + antidepressants	Antidepressants	15	92.20%	<0.0001	–	**−1.54 (−2.00, −1.09)**	**<0.0001**
Electroacupuncture + antidepressants	Antidepressants	2	87.10%	0.005	–	−0.73 (−1.90, 0.44)	0.222
Eye acupuncture + antidepressants	Antidepressants	1	–	–	–	−0.24 (−0.56, 0.07)	0.128
Moxibustion + antidepressants	Antidepressants	2	0%	0.871	–	**−1.61 (−2.01, −1.21)**	**<0.0001**
Self-Rating Depression Scale score							
Wrist-ankle acupuncture + antidepressants	Antidepressants	1	–	–	–	**−0.92 (−1.36, −0.48)**	**<0.0001**
Acupressure + antidepressants	Antidepressants	1	–	–	–	**−2.61 (−3.11, −2.11)**	**<0.0001**
Auricular pressure + antidepressants	Antidepressants	1	–	–	–	**−2.30 (−2.96, −1.64)**	**<0.0001**
Electroacupuncture + antidepressants	Antidepressants	1	–	–	–	**−0.67 (−1.19, −0.15)**	**0.012**
National Institutes of Health Stroke Scale score							
Moxibustion + antidepressants	Antidepressants	1	–	–	–	**−1.95 (−2.52, −1.37)**	**<0.0001**
Abdominal acupuncture + antidepressants	Antidepressants	2	0%	0.961	–	**−0.62 (−1.03, −0.21)**	**0.003**
Conventional acupuncture + antidepressants	Antidepressants	6	96.60%	<0.0001	–	**−1.11 (−2.12, −0.11)**	**0.029**
Electroacupuncture + antidepressants	Antidepressants	2	68.90%	0.073	–	**−0.82 (−1.41, −0.23)**	**0.007**

Bold indicates statistically significant.

#### 3.4.2. Hamilton Depression Scale score

The results of the meta-analysis showed that electroacupuncture plus antidepressants, abdominal acupuncture plus antidepressants, conventional acupuncture plusantidepressants and moxibustion plus antidepressants were statistically significant differences in HAMD scores compared with antidepressants (*p* < 0.05). While theHAMD scores of auricular electroacupuncture plus antidepressants and electroac-upuncture plus antidepressants were not statistically significant when compared with antidepressants (*p* > 0.05). The results of descriptive analysis showed that HAMD scores of wrist-ankle acupuncture plus antidepressants and auricular pressure plus antidepressants were statistically significant when compared with antidepressants (*p* < 0.05). While the HAMD score of eye acupuncture plus antidepressants was not statistically significant when compared with antidepressants (*p* > 0.05). The results of pairwise meta-analysis are shown in Table 2.

#### 3.4.3. Self-Rating Depression Scale score

The results of descriptive analysis showed that wrist-ankle acupuncture plus antidepressants, acupressure plus antidepressants, auricular pressure plus antidepressants and electroacupuncture plus antidepressants were statistically significant differences in SDS scores compared to antidepressants (*p* < 0.05). The results of pairwise meta-analysis are shown in Table 2.

#### 3.4.4. National Institutes of Health Stroke Scale score

The results of the meta-analysis showed that abdominal acupuncture plus antidepressants, conventional acupuncture plus antidepressants and electroacupuncture plus antidepressants were statistically significant differences in NIHSS scores compared with antidepressants (*p* < 0.05). In contrast, there was no statistically significant difference between the HAMD scores of auricular electroacupuncture plusantidepressants and electroacupuncture plus antidepressants compared with antidepressants (*p* > 0.05). The results of the descriptive analysis showed a statistically significant difference in NIHSS scores for moxibustion plus antidepressants compared to antidepressants (*p* < 0.05). The results of pairwise meta-analysis are shown in Table 2.

### 3.5. Subgroup analysis

To further explore the effect of treatment duration on outcomes, a subgroup analysis was conducted in this study. Due to the small amount of literature involved in some acupuncture treatments, it was impossible to perform subgroup analyses for all outcome indicators. Therefore, only the total efficiency and HAMD scores involved in the conventional acupuncture plus antidepressants group were analyzed in subgroups. Subgroup analysis showed that conventional acupuncture plus antidepressants was statistically different in terms of increasing total effective rate and improving HAMD scores for both treatment duration >6 weeks and treatment duration ≤6 weeks (*p* < 0.05). The results of the subgroup analysis are shown in Supplementary Figures S1, S2.

### 3.6. Sensitivity analysis

Due to the limited number of included literature and the data provided in the literature, sensitivity analysis could not be performed for all outcome indicators. Sensitivity analyses were performed for electroacupuncture plus antidepressants and conventional acupuncture plus antidepressants for total effective rate and HAMD scores, and the results showed stable results in the meta-analysis. For NIHSS scores, sensitivity analysis was performed only for conventional acupuncture plus antidepressants, and the results showed that the meta-analysis was stable. The results of the sensitivity analysis are shown in Supplementary Figures S3–S7.

### 3.7. Results of network meta-analysis

#### 3.7.1. Evidence network diagram

**Figure 2:**
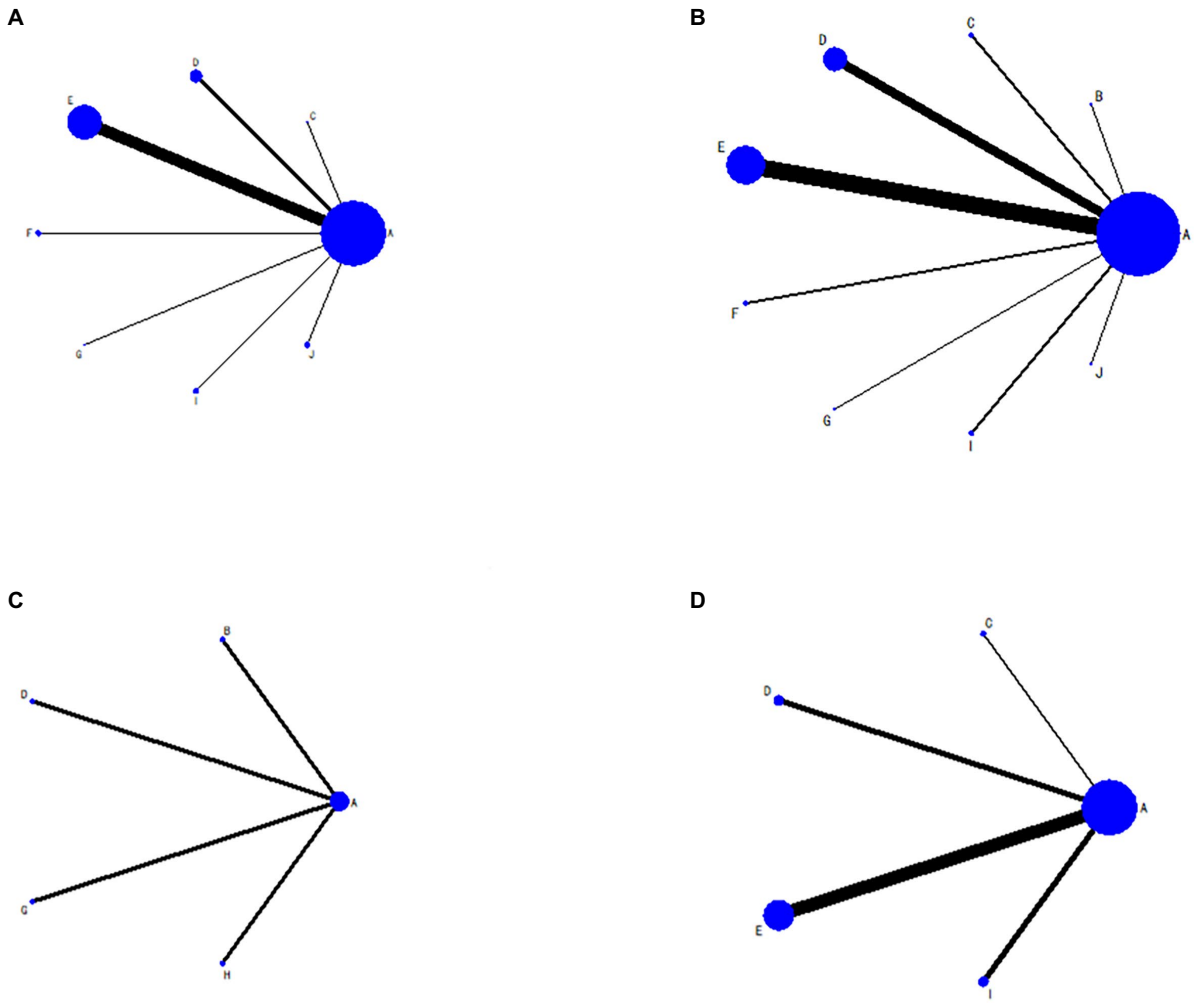
Evidence network diagram. **(A)** Total effective rate; **(B)** Hamilton Depression Scale score; **(C)** Self-Rating Depression Scale score; **(D)** National Institutes of Health Stroke Scale score; (A) antidepressants; (B) wrist-ankle acupuncture plus antidepressants; (C) moxibustion plus antidepressants; (D) electroacupuncture plus antidepressants; (E) conventional acupuncture plus antidepressants; (F) auricular electroacupuncture plus antidepressants; (G) auricular pressure plus antidepressants; (H) acupressure plus antidepressants; (I) abdominal acupuncture plus antidepressants; (J) eye acupuncture plus antidepressants.

The evidence network for each intervention is shown in Figure 2, where the dots represent the interventions included in the analysis and the lines indicate the existence of directly comparable RCTs between the two interventions. The thicker the line between the two interventions, the larger the number of studies, and the larger the dots, the larger the sample size of the studies.

#### 3.7.2. Network meta-analysis results of total effective rate

**Figure 3:**
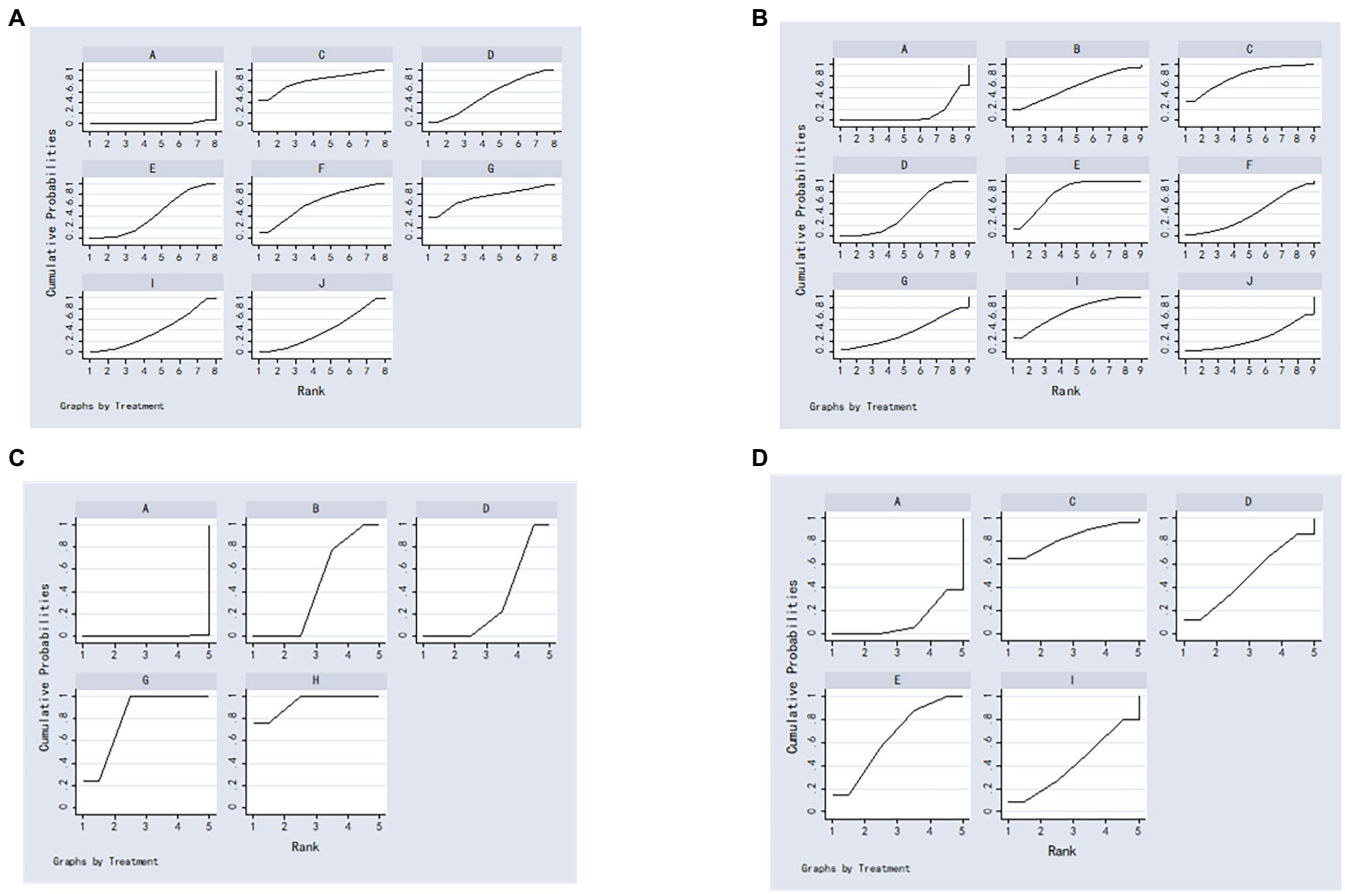
The surface under the cumulative ranking curve plots. **(A)** Total effective rate; **(B)** Hamilton Depression Scale score; **(C)** Self-Rating Depression Scale score; **(D)** National Institutes of Health Stroke Scale score; (A) antidepressants; (B) wrist-ankle acupuncture plus antidepressants; (C) moxibustion plus antidepressants; (D) electroacupuncture plus antidepressants; (E) conventional acupuncture plus antidepressants; (F) auricular electroacupuncture plus antidepressants; (G) auricular pressure plus antidepressants; (H) acupressure plus antidepressants; (I) abdominal acupuncture plus antidepressants; (J),eye acupuncture plus antidepressants.

The results of the NMA showed that moxibustion plus antidepressants, auricular electroacupuncture plus antidepressants, electroacupuncture plus antidepressants, conventional acupuncture plus antidepressants, abdominal acupuncture plus antidepressants and eye acupuncture plus antidepressants had better total effective rate than antidepressants, with no statistically significant differences between the other treatments (Table 3). Ranking results of SUCRA: moxibustion plus antidepressants (78.2) > auricular pressure plus antidepressants (75.1) > auricular electroacupuncture plus antidepressants (65.5) > electroacupuncture plus antidepressants (55.0) > conventional acupuncture plus antidepressants (44.3) > abdominal acupuncture plus antidepressants (40.5) > eye acupuncture plus antidepressants (40.3) > antidepressants (1.1) (Figure 3A).

**Table 3:** Network meta-analysis of total effective rate.

Interventions	RR (96% CI)							
	Moxibustion plus antidepressants	Auricular pressure plus antidepressants	Auricular Electroacupuncture plus antidepressants	Electroacupuncture plus antidepressants	Conventional acupuncture plus antidepressants	Abdominal acupuncture plus Antidepressants	Eye acupuncture plus antidepressants	Antidepressants
Moxibustion plus antidepressants	0							
Auricular pressure plus antidepressants	1.01 (0.71, 1.44)	0						
Auricular electroacupuncture plus antidepressants	1.07 (0.82, 1.41)	1.06 (0.79, 1.43)	0					
Electroacupuncture plus antidepressants	1.11 (0.86, 1.43)	1.09 (0.82, 1.45)	1.03 (0.87, 1.23)	0				
Conventional acupuncture plus antidepressants	1.13 (0.89, 1.44)	1.12 (0.86, 1.46)	1.06 (0.91, 1.22)	1.02 (0.91, 1.15)	0			
Abdominal acupuncture plus Antidepressants	1.15 (0.88, 1.49)	1.13 (0.85, 1.51)	1.07 (0.89, 1.28)	1.04 (0.89, 1.21)	1.01 (0.89, 1.15)	0		
Eye acupuncture plus antidepressants	1.15 (0.88, 1.48)	1.13 (0.85, 1.50)	1.07 (0.89, 1.27)	1.03 (0.89, 1.21)	1.01 (0.89, 1.14)	1.00 (0.85, 1.17)	0	
Antidepressants	**1.30 (1.03, 1.65)**	1.29 (0.99, 1.67)	1.21 (1.06, 1.39)	1.18 (1.06, 1.31)	1.15 (1.09, 1.21)	1.14 (1.01, 1.28)	**1.14 (1.02, 1.27)**	0

Bold indicates statistically significant.

#### 3.7.3. Network meta-analysis results of HAMD score

The results of the NMA showed that conventional acupuncture plus antidepressants, moxibustion plus antidepressants, abdominal acupuncture plus antidepressants, electroacupuncture plus antidepressants and eye acupuncture plus antidepressants had better HAMD scores than antidepressants, and conventional acupuncture plus antidepressants had better HAMD scores than antidepressants. Conventional acupuncture plus antidepressants had better HAMD scores than electroacupuncture plus antidepressants, with no statistically significant differences between the other treatments (Table 4). Ranking results of SUCRA: conventional acupuncture plus antidepressants (78.7) > moxibustion plus antidepressants (78.6) > abdominal acupuncture plus antidepressants (73.2) > wrist-ankle acupuncture plus antidepressants (60.8) > electroacupuncture plus antidepressants (44.9) > auricular electroacupuncture plus antidepressants (41.5) > auricular pressure plus antidepressants (36.7) > eye acupuncture plus antidepressants (25.0) > antidepressants (10.5) (Figure 3B).

**Table 4:** Network meta-analysis of Hamilton Depression Scale score.

Interventions	SMD (96% CI)								
	Conventional acupuncture plus antidepressants	Moxibustion plus antide-pressants	Abdominal acupuncture plus antidepressants	Wrist-ankle acupuncture plus antidepressants	Electroacupuncture plus antidepressants	Auricular electro-acupuncture plus antidepressants	Auricular pressure plus antide-pressants	Eye acupuncture plus antide-pressants	Antidepressants
Conventional acupuncture plus antidepressants	0								
Moxibustion plus antidepressants	0.07 (−1.17, 1.32)	0							
Abdominal acupuncture plus antidepressants	−0.04 (−1.31, 1.22)	−0.12 (−1.79, 1.55)	0						
Wrist-ankle acupuncture plus antidepressants	−0.33 (−2.00, 1.34)	−0.40 (−2.40, 1.59)	−0.28 (−2.29, 1.72)	0					
Electroacupuncture plus antidepressants	**−0.71 (−1.40, −0.01)**	−0.78 (−2.07, 0.51)	−0.66 (−1.97, 0.65)	−0.38 (−2.08, 1.33)	0				
Auricular electroacupuncture plus antidepressants	−0.80 (−2.02, 0.42)	−0.87 (−2.50, 0.76)	−0.75 (−2.40, 0.89)	−0.47 (−2.45, 1.51)	−0.09 (−1.36, 1.18)	0			
Auricular pressure plus antidepressants	−0.97 (−2.66, 0.72)	−1.04 (−3.05, 0.97)	−0.92 (−2.95, 1.10)	−0.64 (−2.94, 1.66)	−0.26 (−1.99, 1.46)	−0.17 (−2.16, 1.82)	0		
Eye acupuncture plus antidepressants	−1.28 (−2.92, 0.36)	−1.35 (−3.32, 0.62)	−1.23 (−3.22, 0.75)	−0.95 (−3.21, 1.31)	−0.57 (−2.25, 1.10)	−0.48 (−2.43, 1.47)	−0.31 (−2.59, 1.97)	0	
Antidepressants	**−1.52 (−1.95, −1.10)**	**−1.59 (−2.76, −0.42)**	**−1.48 (−2.67, −0.29)**	−1.19 (−2.81, 0.42)	**−0.82 (−1.36, −0.27)**	−0.72 (−1.87, 0.42)	−0.55 (−2.19, 1.08)	**−0.24 (−1.83, 1.34)**	0

Bold indicates statistically significant.

#### 3.7.4. Network meta-analysis results of SDS score

The results of the NMA showed that acupressure plus antidepressants, auricular pressure plus antidepressants, wrist-ankle acupuncture plus antidepressants and electroacupuncture plus antidepressants had better SDS scores than antidepressants and acupressure plus antidepressants had better SDS scores than electroacupuncture plus antidepressants and wrist-ankle acupuncture plus antidepressants; SDS scores for auricular pressure plus antidepressants were better than electroacupuncture plus antidepressants and wrist-ankle acupuncture plus antidepressants. There were no statistically significant differences between the other treatments (Table 5). Ranking results of SUCRA: acupressure plus antidepressants (94.1) > auricular pressure plus antidepressants (80.9) > wrist-ankle acupuncture plus antidepressants (44.4) > electroacupuncture plus antidepressants (30.4) > antidepressants (0.2) (Figure 3C).

**Table 5:** Network meta-analysis of Self-Rating Depression Scale score.

Interventions	SMD (96% CI)				
	Acupressure plus antidepressants	Auricular pressure plus antidepressants	Wrist-ankle acupuncture plus antidepressants	Electroacupuncture plus antidepressants	Antidepressants
Acupressure plus antidepressants	0				
Auricular pressure plus antidepressants	−0.32 (−1.15, 0.51)	0			
Wrist-ankle acupuncture plus antidepressants	**−1.68 (−2.34, −1.01)**	**−1.36 (−2.15, −0.57)**	0		
Electroacupuncture plus antidepressants	**−1.93 (−2.65, −1.21)**	**−1.61 (−2.45, −0.77)**	−0.25 (−0.93, 0.43)	0	
Antidepressants	**−2.59 (−3.09, −2.09)**	**−2.27 (−2.93, −1.61)**	**−0.91 (−1.35, −0.48)**	**−0.66 (−1.18, −0.14)**	0

Bold indicates statistically significant.

#### 3.7.5. Network meta-analysis results of NIHSS score

The results of the NMA showed that conventional acupuncture plus antidepressants had better NIHSS scores than antidepressants, with no statistically significant differences between the other therapies (Table 6). Ranking results of SUCRA: moxibustion plus antidepressants (83.2) > conventional acupuncture plus antidepressants (64.4) > electroacupuncture plus antidepressants (49.8) > abdominal acupuncture plus antidepressants (41.6) > antidepressants (10.9) (Figure 3D).

**Table 6:** Network meta-analysis of National Institutes of Health Stroke Scale score.

Interventions	SMD (96% CI)				
	Moxibustion plus antidepressants	Conventional acupuncture plus antidepressants	Electroacupuncture plus antidepressants	Abdominal acupuncture plus antidepressants	Antidepressants
Moxibustion plus antidepressants	0				
Conventional acupuncture plus antidepressants	−0.83 (−3.24, 1.59)	0			
Electroacupuncture plus antidepressants	−1.12 (−3.85, 1.62)	−0.29 (−2.10, 1.52)	0		
Abdominal acupuncture plus antidepressants	−1.32 (−4.06, 1.43)	−0.49 (−2.32, 1.34)	−0.20 (−2.43, 2.03)	0	
Antidepressants	−1.92 (−4.16, 0.32)	**−1.10 (−2.01, −0.19)**	−0.81 (−2.37, 0.76)	−0.61 (−2.19, 0.98)	0

Bold indicates statistically significant.

### 3.8. Small sample effect estimation

**Figure 4:**
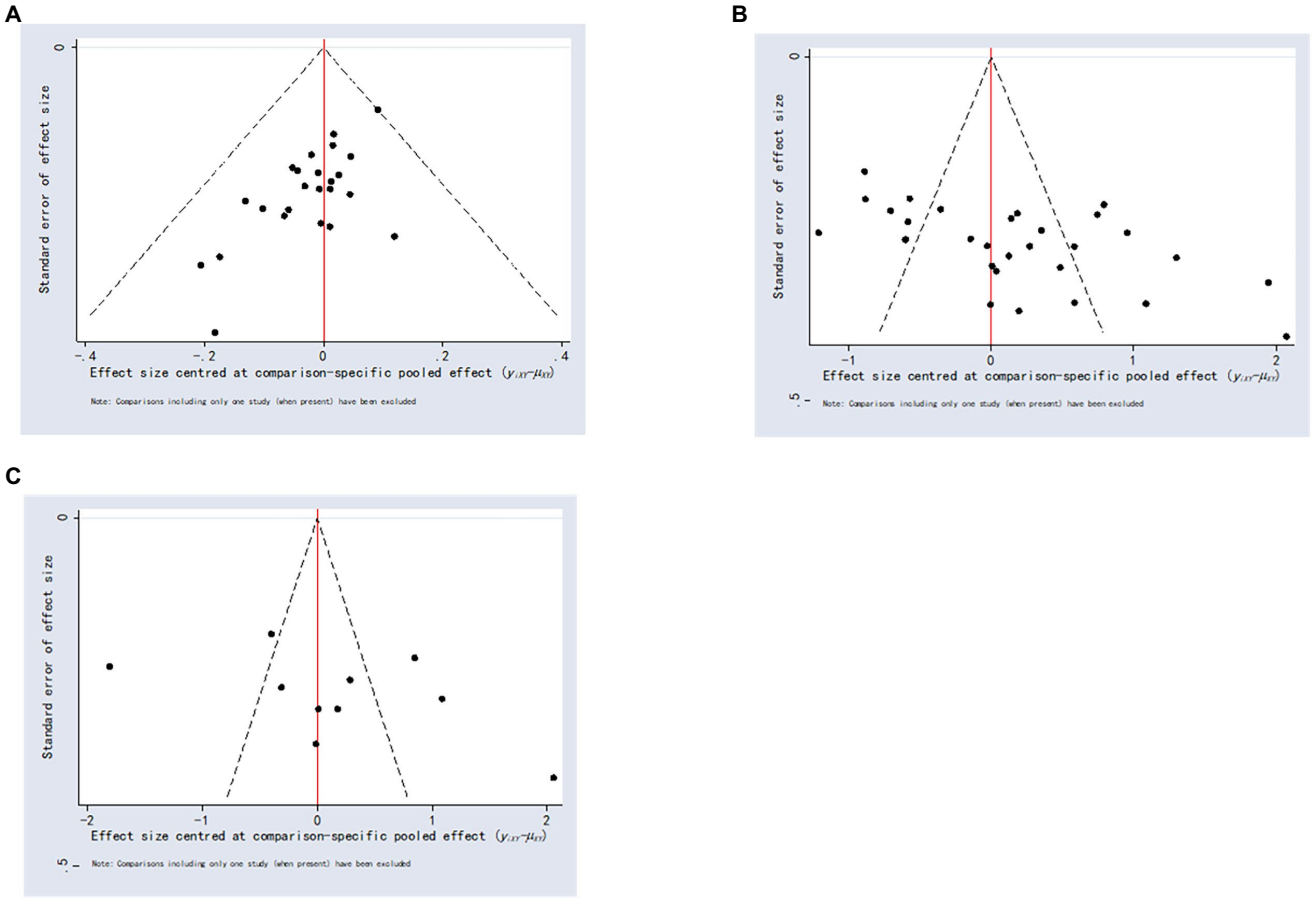
Comparison-correction funnel plot. **(A)** Total effective rate; **(B)** Hamilton Depression Scale score; **(C)** National Institutes of Health Stroke Scale score.

Figure 4 shows the comparison-adjusted funnel plots for the total effective rate, HAMD scores, and HIHSS scores, as the figure shows that the symmetry of the distribution is fair and some studies fall on the outside of the funnel plot, suggesting a possible publication bias or small sample effect.

## 4. Discussion

A total of 2,898 patients with 38 literatures (17–24, 31–60) involving nine acupuncture treatments were included in this study. This study evaluated the effect of acupuncture combined with antidepressants on the total effective rate, HAMD score, NIHSS score and SDS score in patients with PSD. Our results showed that moxibustion plus antidepressants was the most effective in terms of increasing total effective rate, based on the probability ranking results. The NMA results show that the total clinical efficacy rate of moxibustion plus antidepressants, auricular electroacupuncture plus antidepressants, electroacupuncture plus antidepressants, conventional acupuncture plus antidepressants, abdominal acupuncture plus antidepressants and eye acupuncture plus antidepressants were superior to that of antidepressants. In terms of improving HAMD scores, conventional acupuncture plus antidepressants, moxibustion plus antidepressants, abdominal acupuncture plus antidepressants, electroacupuncture plus antidepressants and eye acupuncture plus antidepressants were superior to antidepressants. HAMD scores for conventional acupuncture plus antidepressants was better than electroacupuncture plus antidepressants. Based on the probability ranking results, conventional acupuncture plus antidepressants was the most effective. In terms of reducing SDS scores, acupressure plus antidepressants was the most effective according to the probability ranking results. Furthermore, the NMA results showed that acupressure plus antidepressants, auricular pressure plus antidepressants, wrist-ankle acupuncture plus antidepressants and electroacupuncture plus antidepressants had better SDS scores than antidepressants and acupressure plus antidepressants had better SDS scores than electroacupuncture plus antidepressants and wrist-ankle acupuncture plus antidepressants; SDS scores for auricular pressure plus antidepressants was better than electroacupuncture plus antidepressants and wrist-ankle acupuncture plus antidepressants. In terms of improving NIHSS scores, the probability ranking results showed that moxibustion plus antidepressants was the most effective, while the NMA results showed that conventional acupuncture plus antidepressants had better NIHSS scores than antidepressants. The above results showed that although the efficacy of different indicators varied, according to the results of the study, the top ranking was moxibustion plus antidepressants, conventional acupuncture plus antidepressants and acupressure plus antidepressants.

The pathogenesis of PSD is still unclear and may be related to neurotransmitter theory, cytokine inflammation theory, gene polymorphism theory, psychosocial factors, etc. (61–63). PSD not only hinders the recovery of neurological and cognitive functions of stroke patients, but also causes great mental suffering and seriously affects the quality of life of patients (64, 65). Acupuncture plays an important role in the treatment of PSD as a reliable and safe alternative therapy. Zhang et al. (26) showed that acupuncture combined with antidepressants was superior to antidepressants alone in the treatment of PSD. Both conventional acupuncture combined with antidepressants and electroacupuncture combined with antidepressants showed the same effect. Hang et al.’s (66) NMA results on site-specific acupuncture methods for PSD showed that site-specific acupuncture therapy, the combination of two acupuncture therapies, and the combination of acupuncture and medicine may be effective and safe in improving the condition of patients with PSD. The results of a meta-analysis by Guo et al. (25) showed that moxibustion can change HAMD values and improve the efficiency of PSD treatment. The present study found that conventional acupuncture plus antidepressants, moxibustion plus antidepressants, abdominal acupuncture plus antidepressants, electroacupuncture plus antidepressants and eye acupuncture plus antidepressants had better HAMD scores than antidepressants. Moxibustion plus antidepressants, auricular electroacupuncture plus antidepressants, electroacupuncture plus antidepressants, conventional acupuncture plus antidepressants, abdominal acupuncture plus antidepressants and eye acupuncture plus antidepressants had better total effective rate than antidepressants. The present study adds several acupuncture therapies to the previous one and also confirms the results of the previous meta-analysis. The treatment time of PSD is relatively long, the side effects of antidepressants and the need to take them for a long time will make it difficult for patients to adhere to them, and eventually the efficacy will not be very good (67). Long-term use may lead to drowsiness, general weakness, gastrointestinal and other adverse effects, and easy to develop drug resistance (68). Therefore, in this context, it is important to choose the appropriate combination treatment plan to shorten the treatment duration for the treatment of PSD and to reduce medical resources. Considering this very important clinical issue, a subgroup analysis of the treatment course was performed in this study. Subgroup analyses showed that conventional acupuncture plus antidepressants were statistically different in terms of increasing total effective rate and improving HAMD scores, both for treatment duration >6 weeks and for treatment duration ≤6 weeks. This suggests that conventional acupuncture plus antidepressants can treat PSD in the short term. Sensitivity analysis on the influence of electroacupuncture plus antidepressants and routine acupuncture plus antidepressants on total response rate and HAMD scores showed that the meta-analysis results were stable. For NIHSS scores, sensitivity analysis of routine acupuncture plus antidepressants showed that the meta-analysis was stable. Therefore, this study recommends conventional acupuncture plus antidepressants as a combination regimen for the treatment of PSD.

Modern research has shown that acupuncture can prevent and treat PSD by regulating abnormal neurotransmitter levels, inhibiting inflammatory factor secretion, increasing neurotrophic protein growth factor levels, reducing hippocampal neuron apoptosis, repairing damaged hippocampal neurons, regulating signaling pathway-related protein expression, and inhibiting hypothalamic–pituitary–adrenal axis hypersecretion (16, 69). Moxibustion facilitates antidepressant effects by promoting the brain’s uptake of tryptophan and converting tryptophan metabolism to 5-HT (70). Electroacupuncture can enhance the body’s defense function, inhibit the secretion of inflammatory factors after stroke, and thus reduce the treatment of injury PSD (71). Acupuncture points on the abdomen can stimulate signals to the gut-brain through the abdominal wall, thus exerting the abdominal-brain correspondence regulation mechanism to treat PSD (72). According to the biological holographic theory of auricular acupuncture points, the stimulation of auricular acupuncture points by acupuncture and bean pressure can be conveyed to brain tissues through holographic reflex pathways, thus regulating brain tissue functions and transforming them to normal functions to prevent and treat PSD (73). Scalp acupuncture is used to improve depression through the “Shen” point of the head, which is located in the frontal and parietal projection areas of the body (74, 75). The eye communicates with the meridians and connects the internal and external organs (76). Eye acupuncture is an emerging acupuncture therapy to prevent and treat PSD by acupuncture at specific points around the eyes (77). Acupuncture has a wide range of effects in the treatment of PSD, and its regulation is characterized by multi-system, multi-link, multi-level, and multi-target overall regulation.

Advantages of this study: This study synthesized clinical trial data from all acupuncture treatments to provide evidence of the efficacy and safety of acupuncture combined with antidepressants in the treatment of PSD, rather than studying several single acupuncture treatments done in previous studies. In addition, we recommend conventional acupuncture plus antidepressants as a combination regimen for PSD because it can improve the total effective rate in a short time. Selection of acupuncture points, and the frequency and technique of acupuncture vary a lot, and lack a unified standard. Through literature review of existing RCTs, the following acupuncture points are mainly selected in the treatment of PSD: Baihui point to awake the brain and open the body, calm the heart and calm the mind; Shenting point and Shenmen point to tonify Qi and calm the spirit; Taichong point and Neiguan point to calm the mind, calm the liver and calm the wind, so as to improve the mood of patients.

This study has some limitations. First, many of the included RCTs did not specifically report randomization methods, allocation concealment, and blinding, which affected the ability to test the meta-analysis. Second, the small number of included studies for certain acupuncture therapies may limit the accuracy of the results. Third, the type and dose of antidepressants, acupuncture point taking, and duration of acupuncture therapy reported in the literature vary, which may increase clinical heterogeneity. Fourth, meta-analysis suffers from some publication bias and small sample effects. Fifth, the limited number of original studies makes it impossible to compare the efficacy of all acupuncture therapies.

In summary, a comparison of the efficacy indicators of the nine different acupuncture therapies combined showed that moxibustion plus antidepressants, conventional acupuncture plus antidepressants and acupressure plus antidepressants are superior in the treatment of PSD. Due to the limitations of the number and quality of included studies, the findings of this study still need to be validated by more high-quality RCTs. Based on the findings of this study, it is suggested that future research should pay attention to: (1) standardize acupuncture point selection treatment standards and standardized operation guidance (angle, depth, manipulation and stimulation amount of acupuncture, etc.) in future research; (2) conduct long-term follow-up to judge the long-term curative effect and recurrence of the disease; (3) carry out RCTs of direct comparison between different acupuncture therapies.

## Data availability statement

The original contributions presented in the study are included in the article/Supplementary material, further inquiries can be directed to the corresponding author.

## Author contributions

CL and MS: conceptualization, software, writing–original draft, and writing—review and editing. SC, SL, and YM: data collection. MS: funding acquisition, resources, and supervision. All authors contributed to the article and approved the submitted version.

## Funding

This work was supported by the Scientific Research Project of Sichuan Provincial Health Commission (20PJ261).

## Conflict of interest

The authors declare that the research was conducted in the absence of any commercial or financial relationships that could be construed as a potential conflict of interest.

## Publisher’s note

All claims expressed in this article are solely those of the authors and do not necessarily represent those of their affiliated organizations, or those of the publisher, the editors and the reviewers. Any product that may be evaluated in this article, or claim that may be made by its manufacturer, is not guaranteed or endorsed by the publisher.

## Abbreviations

PSD: post-stroke depression
NMA: network meta-analysis
PRISMA: Systematic Reviews and Meta-Analyses
RCT: randomized controlled trial
HAMD: Hamilton Depression Scale
NIHSS: National Institutes of Health Stroke Scale
SDS: Depression Self-Rating Scale
RR: Risk ratio; CI, Confidence intervalSMD, Standardized mean difference; SUCRA, Surface under the cumulative ranking curve
