# Supplementary material for: Effect of acupuncture combined with antidepressants on post-stroke depression: A network meta-analysis of nine acupuncture therapy
Source: Front Neurol. 2023 Mar 23;14:979643. doi: 10.3389/fneur.2023.979643 (PMC10076680; doi:10.3389/fneur.2023.979643)
Supplement: Supplementary file 1 [file Data_Sheet_1.docx]

| **Table S1.Pubmed search strategy** | |
| --- | --- |
| **Number** | **Search terms** |
| #1 | Stroke[MeSH] |
| #2 | Apoplexy[Title/Abstract] |
| #3 | Cerebral Stroke[Title/Abstract] |
| #4 | Cerebrovascular Accident[Title/Abstract] |
| #5 | Cerebrovascular Apoplexy[Title/Abstract] |
| #6 | Vascular Accident, Brain[Title/Abstract] |
| #7 | CVA (Cerebrovascular Accident)[Title/Abstract] |
| #8 | Cerebrovascular Accident, Acute[Title/Abstract] |
| #9 | Cerebrovascular Stroke[Title/Abstract] |
| #10 | Stroke, Acute[Title/Abstract] |
| #11 | Acute Cerebrovascular Accident[Title/Abstract] |
| #12 | Acute Cerebrovascular Accidents[Title/Abstract] |
| #13 | Acute Stroke[Title/Abstract] |
| #14 | Acute Strokes[Title/Abstract] |
| #15 | Apoplexy, Cerebrovascular[Title/Abstract] |
| #16 | Brain Vascular Accident[Title/Abstract] |
| #17 | Brain Vascular Accidents[Title/Abstract] |
| #18 | CVAs (Cerebrovascular Accident)[Title/Abstract] |
| #19 | Cerebral Strokes[Title/Abstract] |
| #20 | Cerebrovascular Accidents[Title/Abstract] |
| #21 | Cerebrovascular Accidents, Acute[Title/Abstract] |
| #22 | Cerebrovascular Strokes[Title/Abstract] |
| #23 | Stroke, Cerebral[Title/Abstract] |
| #24 | Stroke, Cerebrovascular[Title/Abstract] |
| #25 | Strokes[Title/Abstract] |
| #26 | Strokes, Acute[Title/Abstract] |
| #27 | Strokes, Cerebral[Title/Abstract] |
| #28 | Strokes, Cerebrovascular[Title/Abstract] |
| #29 | Vascular Accidents, Brain[Title/Abstract] |
| #30 | OR/1-29 |
| #31 | Depression[MeSH] |
| #32 | Depressive Symptoms [Title/Abstract] |
| #33 | Emotional Depression [Title/Abstract] |
| #34 | Depression, Emotional [Title/Abstract] |
| #35 | Depressions [Title/Abstract] |
| #36 | Depressions, Emotional [Title/Abstract] |
| #37 | Depressive Symptom [Title/Abstract] |
| #38 | Emotional Depressions [Title/Abstract] |
| #39 | Symptom, Depressive [Title/Abstract] |
| #40 | Symptoms, Depressive [Title/Abstract] |
| #41 | OR/31-40 |
| #42 | Acupuncture [MeSH] |
| #43 | Acupuncture [Title/Abstract] |
| #44 | Pharmacopuncture [Title/Abstract] |
| #45 | Electroacupuncture [Title/Abstract] |
| #46 | Warm needle [Title/Abstract] |
| #47 | Fire needle [Title/Abstract] |
| #48 | Blood-letting therapy [Title/Abstract] |
| #49 | Warm acupuncture [Title/Abstract] |
| #50 | Fire acupuncture [Title/Abstract] |
| #51 | Moxibustion [MeSH] |
| #52 | Moxibustion [Title/Abstract] |
| #53 | Auricular application pressure [Title/Abstract] |
| #54 | Auricular needle [Title/Abstract] |
| #55 | Acupoint catgut embedding [Title/Abstract] |
| #56 | Acupoint injection [Title/Abstract] |
| #57 | Scalp acupuncture [Title/Abstract] |
| #58 | Scalp needle [Title/Abstract] |
| #59 | Scalp electroacupuncture [Title/Abstract] |
| #60 | Eye acupuncture [Title/Abstract] |
| #61 | Eye needle [Title/Abstract] |
| #62 | Abdominal acupuncture [Title/Abstract] |
| #63 | Abdominal needle [Title/Abstract] |
| #64 | Ear acupuncture [Title/Abstract] |
| #65 | Ear needle [Title/Abstract] |
| #66 | Auricular needle [Title/Abstract] |
| #67 | Auricular acupuncture [Title/Abstract] |
| #68 | Acupoint [Title/Abstract] |
| #69 | Acupressure [Title/Abstract] |
| #70 | Auricular pressure [Title/Abstract] |
| #71 | OR/42-70 |
| #72 | #30 AND #41 AND #71 |

| **Table S2. Overall risk of bias assessment for all included trials** | | | | | | |
| --- | --- | --- | --- | --- | --- | --- |
| Study | Randomisation | Deviations from intervention | Missing outcome data | Measurement of the outcome | Selection of the reported results | Overall risk of bias |
| You Yanli 2020 | Low | Low | Low | Low | Low | Low |
| Zhang Ting 2016 | Some concerns | Some concerns | Low | Low | Low | Some concerns |
| Liu Songlin 2019 | Low | Some concerns | Low | Low | Low | Some concerns |
| Huang Shile 2014 | Low | Low | Low | Low | Low | Low |
| Liu Yuhong 2016 | Low | Some concerns | Low | Low | Low | Some concerns |
| Guo Aisong 2011 | Some concerns | Low | Low | Low | Low | Some concerns |
| Sun Baomin 2010 | Low | Some concerns | Low | Low | Low | Some concerns |
| Peng Huiyuan 2009 | Low | Some concerns | Low | Low | Low | Some concerns |
| Li Li 2011 | Some concerns | Low | Low | Low | Low | Some concerns |
| Zhang Rui 2017 | Low | Some concerns | Low | Low | Low | Some concerns |
| Jiao Haifeng 2012 | Some concerns | Some concerns | Low | Low | Low | Some concerns |
| Wang Zhonghua 2008 | Some concerns | Some concerns | Low | Low | Low | Some concerns |
| Tong Xin 2012 | Some concerns | Some concerns | Low | Low | Low | Some concerns |
| JiaoYonggang 2018 | High | Some concerns | Low | Low | Low | High |
| Shao Hongwei 2020 | Low | Some concerns | Low | Low | Low | Some concerns |
| Duan Jun 2022 | Low | Some concerns | Low | Low | Low | Some concerns |
| Zhang Yongchao 2020 | Low | Some concerns | Low | Low | Low | Some concerns |
| Bi Xueqi 2017 | High | Some concerns | Low | Low | Low | High |
| Zhang Ekeng 2016 | High | Some concerns | Low | Low | Low | High |
| Wang Jingxin 2019 | Low | Some concerns | Low | Low | Low | Some concerns |
| Liu Tai 2016 | Low | Low | Low | Low | Low | Low |
| Zhang Shengli 2016 | Some concerns | Some concerns | Low | Low | Low | Some concerns |
| Xu Jinping 2008 | Some concerns | Some concerns | Low | Low | Low | Some concerns |
| Huang Chunyuan 2013 | Some concerns | Some concerns | Low | Low | Low | Some concerns |
| Zhang Qian 2015 | Low | Some concerns | Low | Low | Low | Some concerns |
| Zhou Yafen 2014 | Low | Low | Low | Low | Low | Low |
| Duan Xiaojing 2012 | Low | Some concerns | Low | Low | Low | Some concerns |
| Jiang Lan 2019 | High | Some concerns | Low | Low | Low | High |
| Xu Changmin 2019 | Low | Some concerns | Low | Low | Low | Some concerns |
| Li Chuanyou 2021 | Low | Some concerns | Low | Low | Low | Some concerns |
| Jian Rui 2020 | Low | Some concerns | Low | Low | Low | Some concerns |
| Zhang Xiaodong 2019 | Some concerns | Some concerns | Low | Low | Low | Some concerns |
| Yan Changchang 2018 | Low | Some concerns | Low | Low | Low | Some concerns |
| Han Yuhui 2018 | Low | Some concerns | Low | Low | Low | Some concerns |
| Qi Linjing 2019 | High | Some concerns | Low | Low | Low | High |
| Xiao Wei 2011 | Low | Some concerns | Low | Low | Low | Some concerns |
| Liu Dan 2013 | Low | Some concerns | Low | Low | Low | Some concerns |
| Zhu Fengkui 2010 | Some concerns | Some concerns | Low | Low | Low | Some concerns |

Figure S1.Subgroup analysis forest plot of total effective rate (Conventional acupuncture plus antidepressants).

Figure S2.Subgroup analysis forest plot of Hamilton Depression Scale score (Conventional acupuncture plus antidepressants).

Figure S3. Sensitivity analysis for total effective rate(Electroacupuncture plus antidepressants vs antidepressants).

Figure S4. Sensitivity analysis for total effective rate(Conventional acupuncture plus antidepressants vs antidepressants).

Figure S5. Sensitivity analysis for Hamilton Depression Scale score(Electroacupuncture plus antidepressants vs antidepressants).

Figure S6. Sensitivity analysis for Hamilton Depression Scale score(Conventional acupuncture plus antidepressants vs antidepressants).

Figure S7. Sensitivity analysis for National Institutes of Health stroke scale score(Conventional acupuncture plus antidepressants vs antidepressants).
